# Policy principles for sustainable and just land systems

**DOI:** 10.1098/rsos.250810

**Published:** 2025-10-15

**Authors:** Rachael Garrett, Patrick Meyfroidt, Ariane de Bremond, Ariani Wartenberg, Lindsay Barbieri, Álvaro Fernández-Llamazares, Emmanuel Acheampong, Thomas Addoah, Matthew Adeleye, Peter Alexander, Joyce Brandão, David Anthony Coomes, Erle C. Ellis, J. Fajardo, Johanna Jacobi, Melissa Leach, Sharachchandra Lele, Aymara Llanque Zonta, Joss Lyons-White, Adrian Martin, Peter Messerli, E. J. Milner-Gulland, Daniel Müller, Morena Mills, Pauline Nantongo Kalunda, Unai Pascual, Ximena Rueda, Casey Ryan, Siddappa Setty, Thu Thuy Pham, Cecilia Zagaria

**Affiliations:** ^1^ Department of Geography, University of Cambridge, Cambridge CB2 1DB, UK; ^2^ Conservation Research Institute, University of Cambridge, Cambridge CB2 3QZ, UK; ^3^ Global Land Programme, College Park 20740, USA; ^4^ Earth and Life Institute, Universite Catholique de Louvain, Louvain-la-Neuve 1348, Belgium; ^5^ Fonds de la Recherche Scientifique, F.R.S.-FNRS, Brussels, 1000, Belgium; ^6^ Department of Geographical Sciences, University of Maryland College Park, College Park 20740, USA; ^7^ Department of Animal Biology, Plant Biology and Ecology (BABVE), Universitat Autònoma de Barcelona, Barcelona 08193, Spain; ^8^ Institute of Environmental Science and Technology (ICTA-UAB), Universitat Autònoma de Barcelona, Barcelona 08193, Spain; ^9^ Department of Silviculture and Forest Management, Kwame Nkrumah University of Science and Technology (KNUST), Kumasi, Ghana; ^10^ School of GeoSciences, University of Edinburgh, Edinburgh EH8 9XP, UK; ^11^ Global Academy of Agriculture and Food Security, University of Edinburgh, Edinburgh EH25 9RG, UK; ^12^ Department of Plant Sciences, Cambridge University, Cambridge CB2 3EA, UK; ^13^ Geography and Environmental Systems, University of Maryland Baltimore County, Baltimore, MD 21250, USA; ^14^ Department of Environmental Systems Science, ETH Zürich, Zürich 8006, Switzerland; ^15^ Department of Social Anthropology, University of Cambridge, Cambridge CB2 3RF, UK; ^16^ Cambridge Conservation Initiative, Cambridge, CB2 3QZ, UK; ^17^ Centre for Environment and Development, Ashoka Trust for Research in Ecology and the Environment, Bengaluru 560064, India; ^18^ Helmholtz Centre for Environmental Research (UFZ), Leipzig 04318, Germany; ^19^ Faculty of Sustainability, Leuphana University, 21335, Germany; ^20^ School of Global Development, University of East Anglia, Norwich NR4 7TJ, UK; ^21^ Wyss Academy for Nature, University of Bern, Bern 3011, Switzerland; ^22^ Department of Biology, Oxford University, Oxford OX1 3EL, UK; ^23^ Leibniz Institute of Agricultural Development in Transition Economies (IAMO), Halle (Saale) 06120, Germany; ^24^ Geography Department, Humboldt-Universität zu Berlin, Berlin 10099, Germany; ^25^ Integrative Research Institute for Transformations in Human-Environment Systems, Humboldt-Universitat zu Berlin, Berlin 12489, Germany; ^26^ Centre for Environmental Policy, Imperial College London, London SW7 1NE, UK; ^27^ Environmental Conservation Trust of Uganda (ECOTRUST), Kampala, Uganda; ^28^ Basque Center for Climate Change, Leioa 48940, Spain; ^29^ Ikerbasque, Basque Foundation for Science, Bilbao, 48009, Spain; ^30^ School of Management, Universidad de los Andes, Bogotá 111711, Colombia; ^31^ College of Business, Government and Law, Flinders University, Bedford Park 5042, Australia; ^32^ Center for International Forestry Research (CIFOR), Bogor, 16115, Indonesia; ^33^ Farming Systems Ecology Group, Wageningen University and Research, Wageningen, The Netherlands

**Keywords:** governance, science–policy, sustainability transitions, conservation, climate, food, transformation

## Abstract

Land systems are the nexus of many global sustainability and justice challenges. Here we present eight guiding principles (P1–8) for improved land system policies following the heuristic stages of a policy cycle. The principles are as follows: embrace recognitional justice (P1), be politically strategic (P2), consider multiple policy goals (P3), address systemic issues (P4), take an integrative scope (P5), foster co-development (P6), adopt clear and monitorable targets (P7) and integrate diagnostic and adaptive capacities (P8). We then explore how well policies align with these principles in two globally relevant cases (land-based climate mitigation and biodiversity-friendly agriculture). In both cases, we find that when policies align poorly with the principles at the agenda-setting stage, there is further misalignment at the policy formulation stage. In the instances when recognitional justice is embraced at the onset, policies subsequently integrate more diverse goals and co-development, but they insufficiently consider political strategy and struggle to handle system complexity. Nonetheless, we identify promising policy mixes that provide benefits to multiple actors, integrate multiple goals, take an integrative scope and have strong monitoring and adaptation, aligning well with multiple principles. Further investigation of these principles could reveal promising policy pathways for land systems.

## 1. Introduction

Achieving greater sustainability and justice of land systems is critical for addressing intersecting biodiversity, livelihood, food and energy security and climate challenges from local to global scales. Yet the complexity of how we use land is often ignored in the rush towards ‘silver bullet’ solutions to these challenges [1–3]. We can see this in unbridled enthusiasm for specific land-use interventions, like tree planting and other ‘nature-based solutions’ to climate change, as well as in growing international interest in particular policy levers, like carbon and biodiversity offset schemes, payments for ecosystem services and nature-related green bonds.

This high attention focus on singular ‘solutions’ is problematic due to inherent features of land systems and the people within them, including historical injustices, value plurality, power asymmetries and system complexity [4–7]. Land systems and their changes are characterized by numerous feedbacks, spillovers and thresholds that lead to path dependencies and irreversibilities [4,8]. These are exacerbated by systemic features of land systems that tend to lock in certain socio-technical and market paradigms, like agricultural commodity production [9]. Therefore, efforts to intervene in one part of a land system very often have reverberations affecting other parts of the system or land systems elsewhere. For this reason, there have been ongoing calls in land systems science, as well as historically in natural resource governance, to avoid ‘panacea’ thinking and focus more on policy mixes than individual levers [10–13].

Land holds multiple meanings and values to different actors, and the vast majority of land globally is already inhabited, used or designated for some purpose [4,14–16]. This implies that most contemporary changes to the land system involve trade-offs between different stakeholders and rightsholders. Therefore, both interventions into land systems and their outcomes tend to be contested [17,18]. This is especially true when the interventions focus on addressing symptoms of land system unsustainability and not their root causes, such as rising consumption, disconnection from nature, biased institutions and unequal societal structures [19]. Less powerful actors often lose out unless interventions and policy changes are specifically justice-centred and aim to address the systemic inequities driving current top-down land-use decisions and their outcomes [20,21]. Although efforts have been made to incorporate these power imbalances—such as the principle of Free, Prior and Informed Consent, enshrined in the UN Declaration on the Rights of Indigenous Peoples, or the suite of safeguards adopted by financial institutions investing in land-related projects—their implementation of such policies is rarely adequate or complete. Calls for transformative change, i.e. efforts to promote system-wide reconfiguration of societal structures, seek to address these trade-offs and resulting injustices [22] by tipping the system into new pathways [23–25].

But what policy approaches can help achieve simultaneous calls for sustainability, justice and transformation in land systems? Here, we propose a checklist of guiding principles to improve policymaking around land systems. Our checklist aims to support government officials and actors working at the science–policy interface, but also contributes to academic understanding and debate about the features guiding good governance in socio-ecological systems. In diagnosing the limitations of existing sustainability policy approaches, our perspective builds on other recent conceptual papers grappling with sustainability governance in a plural and unequal world (e.g. Meyfroidt *et al.* [4] and Pascual *et al.* [23]). We respond to many of the challenges raised by these authors by integrating broader ideas about how to manage wicked and contentious problems [22,26,27], including considerations of political strategy and policy processes. In following a general ‘policy cycle’ approach (from agenda setting to policy formulation, implementation, evaluation and adaptation), our checklist involves some temporality, while acknowledging that cycles necessarily have feedback, and the timing of each element is heuristic rather than definitive. All parts of the cycle must be considered from the outset and continuously revisited.

After presenting the elements of the checklist, we then examine the extent to which existing policies align with the guiding principles using the examples of land-based climate mitigation and biodiversity-friendly agriculture policies. The complexity of issues and contexts in land systems make a comprehensive review of sustainability and justice outcomes across existing land policies difficult. We instead offer an exploratory comparative case study approach to examine two similar, globally relevant cases. We explore the alignment between the policies addressing these challenges and our guiding principles. The cases are similar in that they are heavily influenced by internationally relevant environmental targets and involve complex socio-ecological processes with many potential trade-offs, synergies, and telecouplings.

## 2. Methods

The checklist was developed through expert consultation techniques, combining theory from the literature with the logic and experience of 22 academics and science communicators from 11 countries to develop principles about how land system policy design can lead to more sustainable and just outcomes. The process of developing this article first involved a Royal Society-funded workshop in Cambridge, UK, to identify the high-level principles for land systems policy and explore them through two prominent land system cases (land-based climate mitigation and biodiversity-friendly farming). Participants in the original workshop (all of whom were invited to become co-authors) brought diverse experience, knowledge and backgrounds and various levels of embeddedness in land system science and policy. The group was selected with the following aims, keeping in mind budget constraints and environmental impacts, which meant that half of the participants should be from Europe: (i) even gender split; (ii) diverse representation outside of Europe; (iii) diverse thematic expertise on different land issues; (iv) experience with interdisciplinarity; and (v) a balance of career stages. The resulting group did not match these aims perfectly, given some cancellations, but otherwise our objectives were met (table 1, column 2). People unable to make the workshop were invited to contribute to the paper (table 1, column 3).

**Table 1.** Summary statistics on participants of the workshop (*n* = 26) and paper (*n* = 31).

participant characteristics	% of workshop participants	% of paper participants
female	46	48
region		
Africa	15	13
Asia	8	10
Europe	54	52
Central & South America	12	10
North America	12	16
Oceania	0	3
have expertise on land-based climate mitigation	77	77
have expertise on biodiversity-friendly agriculture	58	58
early or mid-career	46	48

We then, as a group, elaborated the theoretical constructs and examples underlying the principles, refined the steps further and established criteria for evaluating existing land system policies against the steps. Below, we describe and justify these steps before exploring their implications in the two cases. Feedback from multiple rounds of teaching about and discussing the policy roadmap with policymakers and practitioners helped refine our approach.

## 3. Policy principles for land system sustainability and justice

**Figure 1.**
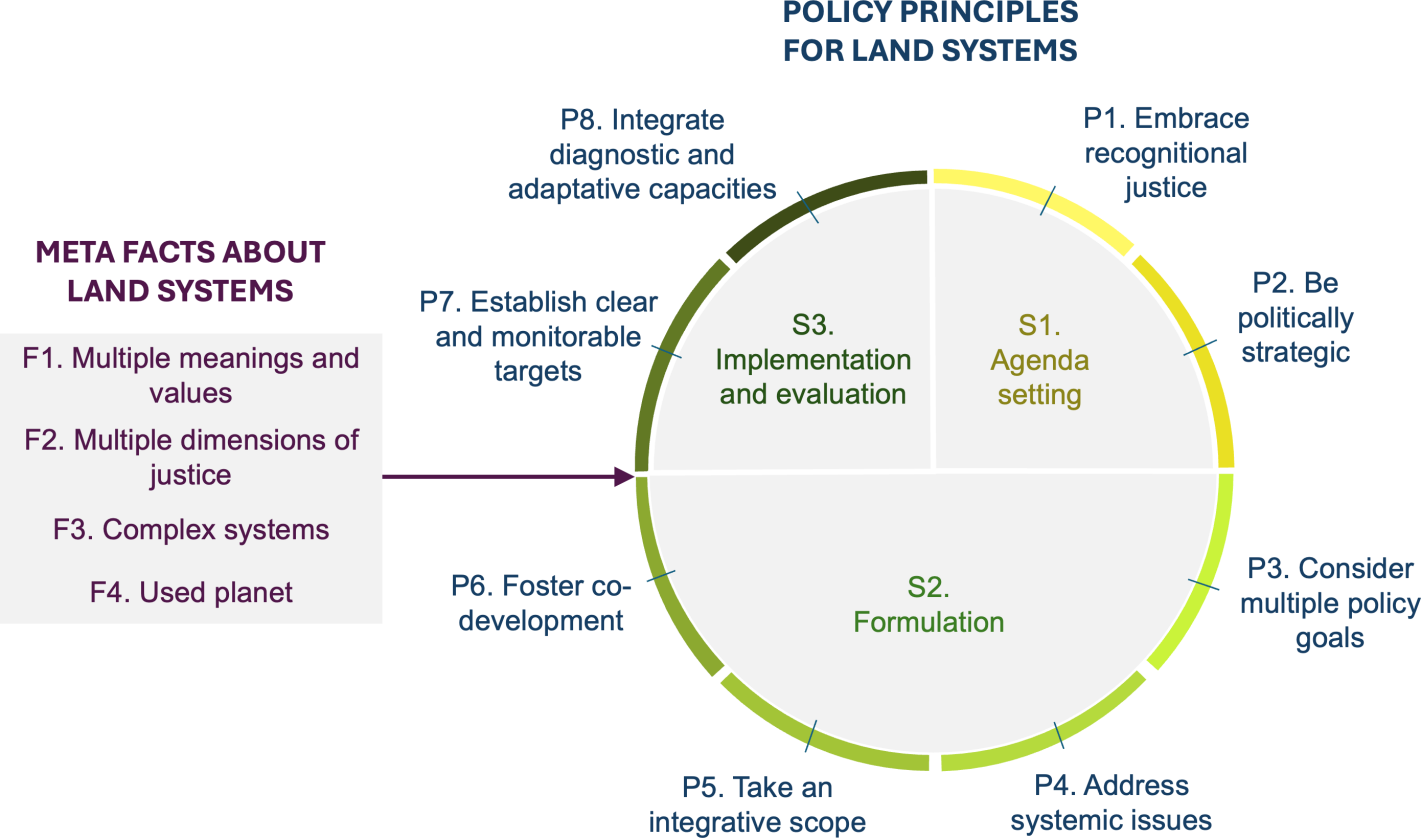
Eight principles for sustainable and just land-use policies (P1-8) along a policy cycle (S1-3) arising from the meta facts about land systems (F1-4)(Meyfroidt *et al.* [4]).

In the following section, we describe and justify the eight policy principles for actors working at the science–policy interface (including scientists and non-governmental entities making recommendations). The principles (P1−8) are summarized in table 2 as a checklist. A full list of the associated literature from which each principle was derived is in the electronic supplementary material, table S1. The principles are organized around the concept of policy cycles, which describes stages in a policy process (figure 1). The first stage of a policy cycle is agenda setting, the process of determining which problems are recognized and deemed as most salient. The next step is policy formulation, involving the definition of objectives and scope to address the problems deemed important. After formulation, policies must be implemented, with specific decision-making rules, codified into codes of conduct and operation (for the private sector) or legislation (for the public sector). Finally, the policy process includes evaluation stages, which can result in adaptation of the policy. While these cycles were initially portrayed as chronological, critiques acknowledge that elements of the cycle have ongoing feedback and current policies are affected by past policies leading to policy layering, so the concept’s utility is more heuristic than deterministic.

**Table 2.** Checklist for developing more sustainable and just land-use policies.

agenda setting			
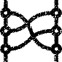	embrace recognitional justice	listen to and acknowledge stakeholders and rightsholders to understand their views and knowledge systems and take past mistakes and historical relationships between policymakers and stakeholders and rightsholders into account	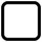
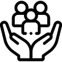	be politically strategic	foresee and mitigate sources of political resistance by combining benefits for multiple stakeholders and rightsholders and enabling small wins and experiences to build momentum for future actions	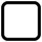

formulation			
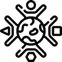	consider multiple policy goals	include justice, rights and diversity as goals of the policy, not just economic efficiency or effectiveness in a single domain	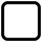
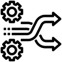	address systemic issues	aim to address the root causes of unsustainable and unjust land systems that contribute to existing trade-offs, including practices, as well as institutional and other societal structures	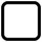
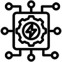	take an integrative scope	include multiple target units, scales and pathways of influence to mitigate potential undesirable spillovers and indirect effects	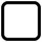
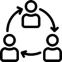	foster co-development	work directly with stakeholders and rightsholders, as well as across sectors to design, implement and adapt policies	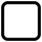

implementation and evaluation			
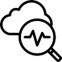	establish clear and monitorable targets	establish clear and monitorable targets given existing capacities and embed tools to monitor these changes	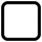
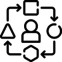	integrate diagnostic and adaptive capacities	build in mechanisms to enable evaluation and shifts in implementation in response to changing outcomes and conditions	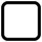

Note: Images are from Flaticon.com (authors: uniconlabs, juicyfish, mynamepong, noomtah, pojok d, freepik, Rizki Ahmad Fauzi).

For land system policies to result in justice and sustainable outcomes, we argue that the agenda-setting phase must start by *embracing recognitional justice* (P1), including by listening to a wide range of stakeholders and rightsholders to understand their views and knowledge systems and acknowledging past mistakes and historical relationships with policymakers. Next, the agenda-setting process should *be politically strategic* (P2), so that the selection of problems and solutions already involves foreseeing and mitigating sources of political resistance by combining benefits for multiple stakeholders and rightsholders and enabling small wins to build momentum for future actions.

At the policy formulation stage, we argue that policymakers and policy brokers should break out of path-dependencies in how we conceptualize ‘good’ and ‘bad’ policies to *consider a greater variety of policy goals* (P3), including justice, rights and diversity, not just economic efficiency or effectiveness in a single domain. In the next step, which relates to calls for transformative change, we encourage policy actors to *address systemic issues* (P4)—which are the root causes of unsustainable and unjust land systems that contribute to existing trade-offs, including practices, as well as institutional and other societal structures—rather than just addressing symptoms. Complex system thinking also entails that policies should (P5) include an *integrative scope* that encompasses different target populations (within and across generations), spatial units and economic sectors. At the formulation stage, we also call for *co-development* (P6), working with local communities and rightsholders as well as working collaboratively across public, private and civil society sectors on formulating the goals, scope and decision-making processes (this principle also extends to implementation). Following from P2, foreseeing the likelihood of discord and building in robust and democratic value negotiation mechanisms is vital [26].

The final principles are focused on improving land system sustainability in the context of complexity, including how to achieve greater adaptability in policy and its implementation, and in the scope of the system being considered. This will help to reduce negative spillovers and indirect effects. Actors involved in the policy process should establish *clear and monitorable definitions and targets* (P7) given existing capacities and embed *monitoring tools* that enable shifts in implementation in response to changing contexts and structural conditions. Actors should then integrate *capacities to diagnose outcomes and challenges and adapt* implementation (P8), should it turn out the policy needs adjusting.

### 3.1. Stage 1: Agenda setting

#### 3.1.1. Embrace recognitional justice

Before defining the problems to be addressed by a policy or possible solutions, it is essential to consider both historical and current relationships between people and the land and how these interconnections shape and are shaped by current and historical relationships between actors. Policies that focus, for example, on optimizing biodiversity, carbon or food production outcomes may ignore the heterogeneous desires, histories, characteristics and beliefs of the land-use actors that will be affected by the policy. These ‘global’ demands on land (e.g. for climate and food security) are heavily skewed towards the views of the Global Minority (white people and other communities benefiting from entrenched power structures) and align with colonial legacies [23]. A key example of this is how Indigenous territories are becoming increasingly exposed to international actors’ desires for carbon sequestration and biodiversity conservation, raising concerns about expanding and intensifying land-use frontiers and elite capture of land-use benefits.

Policymakers should instead seek to understand the socio-cultural and historically embedded perspectives and priorities of land users in any given setting. This includes relational ontological perspectives that are widespread in the Global Majority (people of colour, Indigenous People and traditional communities, ethnic minorities and other members of communities outside entrenched power structures) [28]. The resulting policy mixes should incorporate the ethics of care for, and within, such more-than-human relations [29]. Examples of how to incorporate relationality, reciprocity and interdependence in governance are present in many Indigenous societies [30,31].

Ultimately, this principle calls for going beyond respecting the rights of nature and people to seeing nature and people as mutually constituted in ‘naturekind’ [32], comprising profound interdependencies between humans, non-human animals and plants, as well as their surroundings. Relational thinking contrasts with dominant, dualistic scientific and economic paradigms focused on only instrumental value, i.e. the ecosystem services provided by land or oceans or the conception of places as ‘resources’ [33,34], or intrinsic value, i.e. the inherent value of biodiversity regardless of humans.

Recognition of the perspectives and needs of communities is not only just, but can enable diverse policy and land system actors to identify common challenges and goals [35]. Examples include policies and projects that examine the relationality of communities to places and consider this in their design, as well as initiatives that explicitly acknowledge and address past harms inflicted on communities [36–38]. Better consideration of relationality can, in turn, guide transformative action (below) to better address root causes of issues affecting communities, not just surface-level behaviours [39,40].

#### 3.1.2. Be politically strategic

Policies that challenge existing power structures are likely to encounter high pushback [5,41]. Politically strategic policy-making approaches acknowledge these realities by balancing what is feasible today, while building in wins for different actors to mobilize public and interest group support and reduce political barriers to future policy action [42]. This involves combining policies that involve costs for some actors with those that also bring benefits to the same actors. Politically strategic approaches may also include shifting the narrative from more contentious shifts in values towards more concrete wins. A recent example from the climate policy space focuses on reducing fossil fuel inputs via an integration of carbon taxes that would harm carbon-intensive companies with corporate tax cuts to offset losses to those actors. Proposed policy solutions also include an expansion of investments and job creation in the renewable energy sector [42]. This effort reframed politically charged discussions of pollution and temperature changes that were of limited salience to many communities to a narrative about providing healthy environments and resilient economies for communities, especially those harmed or left behind by extractive economies.

Being politically strategic also includes a temporal dimension. Ideas about ‘ratcheting up’ ambition [43] and ‘incremental change with a transformative agenda’ are forms of policy sequencing that entail having ‘a dual focus on high-level, longer-term transformation combined with an honest recognition of the realities of near-term incrementalism’ [44]. There is some evidence that more ambitious policies often suffer from limited permanence and a ‘ratcheting down’ of ambition [43]. While a desire for transformative change (described below) may prompt a focus on shifting values, beliefs and institutions [45], in some contexts, it may be more effective to build towards these changes alongside policies without advertising them as the primary goals and, instead, focusing on immediate benefits to different groups. An example of this would be the individual market policy instruments introduced to support Indigenous-inspired non-timber forest production and agroforestry in the Amazon biome amidst a broader strategy to support a shift towards justice and a diversity centred bioeconomy [46]. Such an approach provides benefits to multiple actors under current economic structures, e.g. even large industrial farmers can benefit from carbon payments on their farms and supply chain actors can benefit from finance supporting agroforestry efforts. Yet it also paves a way for different economies by showing the success of alternative land uses, building economies of scale, and strengthening alternative actor networks.

Our call for politically strategic policymaking is not the same as focusing on political feasibility or expediency. It will almost always be politically easier to enact policies that serve the interests of status quo actors over those who will benefit from change. Policies must still prioritize the needs of the most marginalized communities and acknowledge, restore and expand their rights. Yet they must also build in benefits for more powerful actors to enhance the tractability of the proposed policies.

### 3.2. Stage 2: Formulation

#### 3.2.1. Include multiple goals

Goals influence the formulation of policy objectives, thus shaping all subsequent policy design decisions and determining flows of benefits and risks associated with policy outcomes [47]. Some policies focus only on *effectiveness*, reaching a singular goal without emphasis on the legitimacy of that goal or the distributional outcomes of the policy [48]. Neoclassical economic approaches to policy design emphasize growth-based economic goals by prioritizing the value of *efficiency*, i.e. the allocation of resources via the market among product end-users in conformity with individual preferences [49]. In these calculations, ‘benefits’ are aggregated into easily comparable, but oversimplified metrics that show how certain resource allocations can maximize net benefits to society. Put in terms of ‘Pareto-efficiency’, this implies a situation in which nobody can be made better off without the theoretical potential to compensate anyone made worse off [50]. When specific outcomes are already pre-defined (e.g. improving access to healthcare or protecting biodiversity), there may be a focus on *cost-effectiveness* instead, i.e. minimizing the costs to achieve a given objective [51]. Taking efficiency and cost-effectiveness as central guiding values generally overlooks policy process considerations, human rights, distributional issues and other justice-based concerns. With a utilitarian focus on net aggregated and easily commensurable benefits, efficiency objectives often reinforce existing inequities in who exactly benefits [6]. A ‘Pareto-optimal’ approach can be highly inequitable, as it does nothing to tackle existing differences in allocations or redistribution of resources or rights to those resources. It can even increase inequality if gains accumulate to already privileged actors, and there is no compensation for losers [52].

Basing policy objectives around *justice*, including procedural (how decisions are made), distributional (how costs and benefits are allocated) and recognitional (how more experiential and subjective elements of dignity, values and identities are addressed) [53] aspects can lead to very different outcomes than those focused on certain stakeholders’ views on effectiveness or efficiency [54]. A focus on restorative justice in particular aims to remove systemic barriers that have historically caused certain groups to incur harm [55] and is thus aligned with transformative change goals. *Diversity* is another distinct goal and in the context of land systems pertains to aiming to have multiple different land covers, uses and management styles, as well as socio-cultural heterogeneity within a landscape. For example, efforts to promote biocultural heritage landscapes, land zoning, agroecological transitions and resilience [35,46,56–58] include a focus on diversity.

Many community resource management and forest rights policies [59] around the world offer good examples of balancing multiple policy goals. These policies clearly include effectiveness goals (i.e. a focus on improving forest health and resources), while embracing both justice and diversity (i.e. by devolving power to manage forests to ethnically and culturally diverse rights-holding communities with high dependencies on forests) to manage forests in ways that are consistent with their world views and experiences. There are numerous other land zoning policies across the world that also embrace multiple goals (e.g. Ecological-Economic Zones in Brazil [60] and Community Resource Management Areas in Ghana [56]).

#### 3.2.2. Address systemic issues

Policies to address land system sustainability challenges are often incremental, i.e. conforming to and accommodating existing economic and political structures and goals [23,47]. For example, creating new one-off market mechanisms to internalize environmental costs, e.g. cap-and-trade systems, payments for ecosystem services and carbon and biodiversity offsets, can tackle a problem temporarily so long as the policy remains in place and the financial incentives are sufficiently high. However, the undesired behaviours are likely to revert as soon as the policy is gone [48]. Since such policies do not address the root causes of the underlying behaviour, low additionality, lack of permanence and leakage are common problems. In fact, many environmental interventions that only focus on proximate causes of environmental harm end up creating ‘off-stage’ (i.e. diffuse, delayed and distant) burdens, not accounted for when designing policy interventions for a given target sector or spatial area [61,62]. Policies that aim to control land users’ behaviours without changing the structures leading to these behaviours often result in trade-offs, injustices and pushback [47].

In contrast, transformative changes call for a fundamental, system-wide reconfiguration of societal structures (e.g. markets, knowledge and power) and institutions (e.g. conventions, norms and rules), which can help reorient seemingly intractable tensions towards co-beneficial outcomes across multiple dimensions or, at least, towards outcomes with fewer trade-offs [63]. A transformative approach to land systems policy necessitates considering the whole coupled human–natural system: changing societal goals for and some land users’ relations to land and to each other (e.g. restoring more healthy relationships), as well as developing new economic models for land (e.g. circular and doughnut economies, socio-bioeconomies [46]) and undertaking efforts that redistribute benefits and empower marginalized and vulnerable groups in society [23,64]. Efforts to simultaneously reduce the negative environmental and health impacts of prevailing food systems by not just improving production sustainability but also tackling biased narratives and advertising campaigns that underpin existing food consumption habits are a good example of a more transformative approach to land systems policies than focusing on sustainable land management alone. On the other hand, it is essential that policies with transformative aims take care to build momentum for larger shifts through incremental benefits to many actors to avoid a future ratcheting down of ambition.

#### 3.2.3. Take an integrative scope

Land systems are complex. Interventions affecting one type of land-use actor in one place almost always have reverberations to other people and places. Yet, many land system sustainability policies only target individual properties or communities (e.g. farm certifications, individual and community payments for ecosystem services, nitrogen taxes, protected area establishment). These approaches ignore interactions between individuals and communities and often fail to achieve the minimum spatial and temporal scales needed for ecological and social benefits [20]. When a policy affects only a single jurisdiction or biome, it often leads to ‘pollution havens’ or ‘sacrifice zones’, which concentrate negative activities in other areas [65,66]. Policies with high stringency (i.e. punitive or reward interventions requiring more behaviour change) may achieve greater results when compliance is high, but often ignore equity and capacity challenges and may be more likely to trigger cheating and leakage of the undesirable land-use activities to other regions, actors or sectors [67,68].

Given these interconnections, it is increasingly recognized that ‘policy mixes’ with an integrative scope, i.e. a combination of multiple policy processes, designs and instruments that cover several dimensions and scales, are a better approach than individual policy levers or projects [67,69,70]. Integrative policy mixes, by including nested scales, overlapping functions and institutional diversity, can help influence more parts of the system at once and with complementary mechanisms (not just incentive-based pathways but also persuasion and information) [71,72]. Policy mixes can also help reconcile effectiveness and equity challenges, since approaches can be adapted to the needs of actors with different needs and constraints.

While policy mixes are necessary to overcome individual scale, sector and instrument limitations, adding more policy instruments is not always better. This myth of ‘additivity’ [26] can hold back more transformative change and add bureaucracy and transaction costs, including burdens on poorer land users. Overlapping policies can create administrative complexity, confusion and conflict [73] and can perversely give rise to ‘institutional shopping’, whereby powerful actors choose the most advantageous institutional framework to advance their own position or evade stricter regulations [74]. Therefore, certain policies may need to be taken away, while other new policies are introduced in a more coherent mix to overcome lock-ins. This can be aided by systematic policy coherence analyses [75,76].

#### 3.2.4. Practise co-development

Policies are usually designed with a variety of inputs—from formal governmental input procedures to community input—but the breadth and depth of input sought for a given policy can look very different depending on the context and scale. Many policies for land systems lack input from local communities—instead, often they are connected to global targets and set by elite actors. Demands for land fall largely on the Global Majority [77] to benefit actors in wealthier and more powerful communities [78]. Beside a lack of process equity in the target design processes, these top-down targets are inherently lacking in local specificity and input. Examples include the United Nations Sustainable Development Goals, the Trillion Trees campaign, 30 by 30 conservation and restoration targets as part of the Kunming Montreal Global Biodiversity Framework, the UNCCD’s Land Degradation Neutrality Targets and the New York Declaration on Forests. The European Union Deforestation Regulation and Nature Restoration Laws also fall into this category [78,79].

These top-down approaches and scientific guidance on how to implement them are often based on global, coarse-grained analyses with no or little input from the communities likely to be affected and may replicate historical oppressive and colonial relationships [20,80]. Important social-ecological contexts and considerations, such as plural values, diverse tenure systems and power inequities, are typically overridden by global concerns, thus widening misalignment between local needs and higher scales of decision-making. A counter-example of how bottom-up targets can be developed is the 2015 Nyélényi Declaration of the International Forum for Agroecology, which was co-developed by different groups of small-scale food producers and consumers, including peasants, Indigenous Peoples and traditional communities, family farmers, rural workers, herders and pastoralists, fisherfolk and urban people [81]. Other examples include policies that devolve policy targets, monitoring and management to communities (e.g. community forestry management approaches in many regions) [82].

Designing policy mixes with inputs from across sectors is also necessary to create coherence and avoid antagonisms [83]. ‘Hybrid’ governance systems combine efforts from multiple actors (public, private and civil society) to address multiple levers at the same time. When actors across different backgrounds can be motivated to work towards complementary rather than substitutive or antagonistic approaches, it tends to yield significantly better results [83].

Co-design and hybrid governance both involve the development of deliberative processes that ensure the right, opportunity and capacity of those who are subject to a decision (or their representatives) to participate in that decision. The opportunity to participate in consequential deliberation means mutual justification and reasoning and refers to non-coercive communication capable of inducing reflection and able to reach actors with different views and mindsets [84]. Citizens’ assemblies and serious games are approaches to co-designing policies that can in principle overcome polarization through deliberation [85]. Roundtables are, in theory, another promising deliberative approach focused on hybrid, voluntary policy development, but, in practice, local communities and Global Majority participants are rarely on an equal footing to large NGOs or corporate players [86,87].

### 3.3. Stage 3: Implementation and evaluation

#### 3.3.1. Establish clear and monitorable targets

Clear definitions are needed to avoid different interpretations of policy rules. A salient example of this can be seen in company-level ‘zero-deforestation’ policies, which tend to ignore national and sub-national differences in definitions of forests [67], leading to contestation over what constitutes a violation of the policy [88]. Similarly, policies relating to ecosystem degradation and restoration rarely give a clear definition for these processes. Defining what the system once was or should be is highly subjective and reflects the perspectives and agendas of particular actors [89]. Yet this subjectivity is rarely acknowledged. Advocating for clarity in definitions is not an argument for aligning behind a single definition for all contexts. Rather, it is about making the differences in definitions across contexts clearer (e.g. as has been done in many certification programmes that tend to have national level interpretations [90]). It is possible to have multiple co-existing definitions for the same concept, so long as each has a clear mechanism for monitoring. Seeking to establish clear but differentiated definitions that are coordinated by an overarching governance system acknowledges the importance of multiple sources of knowledge and context-specificity, akin to a multiple evidence base approach [91], while still providing coherence.

To evaluate policy success, desired outcomes must be able to be monitored, including through impact evaluations [92]. Clarity in definitions (allowing for multiple definitions that can each be monitored) can help improve transparency, but indicator selection and monitoring capacities are also crucial to implementation [93]. The selection of appropriate monitoring indicators also requires the consideration of monitoring capacity at the necessary temporal, thematic and spatial scales and/or intervals. Technological advances (e.g. remote sensing and machine learning) are increasing technical monitoring capabilities rapidly and can help reduce bias in existing monitoring approaches [94]. However, it remains of critical importance to understand limitations, biases and strengths of existing technologies and datasets, as well as indicators, to ensure policy aims are being designed and implemented in sustainable and equitable ways that can also be responsive to changing conditions [82,95]. Carbon accounting in land systems illustrates some of these issues, with inconsistent or incorrect use of terminology for measuring carbon stocks and flows serving as an impediment to understanding the outcomes of interventions [96]. Despite technological advances, monitoring complex carbon cycling processes remains difficult and is compounded by a lack of standardized methods (e.g. sampling protocols for measuring carbon or baseline definitions for calculating additionality) [96,97]. Instead of improving accuracy, poorly designed technological advances can lead to a lack of transparency and undermine local legitimacy [98].

Prioritizing the use of well-defined terms, indicators and monitoring protocols could lead to more clarity and ultimately more successful policy outcomes. Clear, transparent and feasible monitoring mechanisms increase the legitimacy of the policies for all stakeholders, ensuring greater support for their implementation and adaptation [98]. As both implementation and adaptation can be captured by powerful interests, it is necessary that the development of monitoring mechanisms follows co-design processes and sufficiently deliberative governance to integrate plural and relational understandings of the phenomena in question. This process is particularly important as relational values may not be directly comparable or compatible. Deliberation can ensure that relational outcomes are considered.

#### 3.3.2. Integrate diagnostic and adaptive capacities

Monitorability is, in turn, critical to evaluation (assessing impacts) and adaptive governance [99] capacities for continuous adjustments in targets and implementation [63,100]. These processes are needed since land systems are complex with often unpredictable feedback and spillovers. Many well-intentioned top-down policies are excessively rigid, making them unable to adjust to changing circumstances [63]. In such cases, they can end up doing more harm than good (e.g. creating maladaptive outcomes) [101]. Adaptive governance processes focus on learning from the past, foresight, engagement and integration to build capacities and steering mechanisms designed to adjust to changing conditions (e.g. evolving knowledge and technological capacities, changing socio-eco-political contexts and ecosystem states), thus addressing future needs and enhancing resilience [23,102]. Adaptive governance enables learning, experimentation and reflexivity [103,104]. Biocultural approaches that recognize long-term interconnections between culture and biodiversity have often been lauded as good examples of adaptive governance [105] because they explicitly recognize the importance of intergenerational planning and institutions [106], enable social learning [107] and promote flexibility to address complex land-use problems [100,108].

An example of these issues is the case of deforestation in food supply chains. Since 2006, there has been significant civil society pressure and private sector governance focused on targeting a handful of commodities in specific supply chains that are responsible for the greatest amount of deforestation [67,109]. Yet, like many other land system policies, the implementation of zero-deforestation policies targeted at individual suppliers and crops has led to many forms of spillovers to other crops, land users and regions, including the displacement of other food crops to forests [110,111] or production of the same crop in other forest regions [112]. As unintentional outcomes of the original policies, these forms of ‘leakage’ directly undermine the goals of the policy. A failure to incorporate adaptive capacity has locked in a single governance approach that fails to address deforestation adequately, including the proliferation of the same limited approaches in public governance initiatives like the EU Deforestation Regulation. We now explore these issues in more detail in the two cases.

## 4. Exploring the framework in two cases

### 4.1. Case 1: Land-based climate mitigation

Since the early 2000s, land systems have been framed as offering cost-effective solutions to climate change [113], including by avoiding deforestation and restoring ecosystems (or more narrowly planting trees). In recent years, policies supporting bioenergy with carbon capture and storage have also become prominent in international science–policy discussions [114]. Policies in this case are inherently out of alignment with the agenda-setting principles of embracing recognitional justice and being politically strategic, as they originate in neoliberal institutions as a solution to global environmental challenges without prioritizing the interests of the land users or countries most affected by the policies. The perception of many land-based climate mitigation approaches [20] as ‘green grabs’ and the appropriation of nature for environmental ends, associated with dispossession of land and livelihoods, makes them politically unacceptable to many actor coalitions in regions with high carbon stocks. This leads to resistance by the communities that are targeted by the initiatives.

At the formulation stage, conventional climate mitigation policy goals are heavily centred on effectiveness and efficiency values [48,115,116] in meeting these global climate objectives and formulations of specific targets are often unilateral and top-down. Despite the recognition that existing deforestation drivers and restoration barriers are linked to flawed economic paradigms and power relations (e.g. capitalism and neoliberalism), these types of market-focused policies rarely embrace transformative change to address the root causes of ongoing harms. In terms of their scope, some land-based climate mitigation policies focus on multiple scales and actors (e.g. landscape level approaches nested in global finance mechanisms); yet others, including individual protected areas, projects, offsets or supply chain instruments, focus on only a very narrow set of locations and actors. Leakage (i.e. displacement of the negative harms to other regions or actors) is a common problem.

Prominent policies to support avoided deforestation and restoration as climate solutions include payments for environmental services, carbon offset schemes, forest-focused supply chain policies and certifications [117,118]. Such policies are rarely centred on justice or have any alignment with the ethics of care [119] and instead are coupled ad hoc with ‘enabling’ interventions (e.g. outreach, technical assistance, promises of livelihood benefits) to help improve their equity and effectiveness [20,120,121]. These schemes often ignore the values, ontologies and customary rights of affected communities [122] and rarely result in promised benefits to local communities [123]. Protected areas are another approach that can be developed with social justice more at the centre, but, in practice, they are also often top-down, exacerbating social concerns [124,125].

In contrast, policies that focus on the recognition of territorial and community rights are more aligned with ideas of transformative change and can create agency and facilitate movement towards just, equitable and sustainable futures [126,127]. Community-based natural resource management is one approach that is better aligned with co-design, hybrid governance and recognitional justice [82,128]. Indigenous rights recognition processes tend to go further and have been implemented in an emancipatory way (guiding and implementing change towards Indigenous self-determined futures). These processes are built on the recognition that more plural perspectives and worldviews can counter globalization’s hegemonic cultural, political and economic forces [129]. Forms of collective territorial management embrace concepts of prior presence, colonial injustice and relationality, recognizing the historical continuity, ongoing presence and inherent rights of Indigenous Peoples and traditional communities on their territories and the co-evolution of the cultural and biological diversity in these regions [130]. Indigenous and community rights approaches are highly adaptable to different realities and conceptions of management, of making sense of what is their ‘territory’, and the use of their rights as a basis to fight back against encroachment [131].

At the implementation and evaluation stages, both conventional and community-led approaches to address deforestation and foster restoration can be aligned well with clarity and monitorability principles, though with differences. Near-real-time deforestation and disturbance detection is now possible in many places and increasingly aligned with local definitions of forests [132]. New vegetation monitoring capacities are possible with advances in the use of remote sensing (optical, lidar and radar) [133]. These monitoring technologies can be linked to notifications sent to local communities to help them respond quickly to threats and can be integrated with locally relevant, ground-based monitoring. Such combinations can allow for more frequent updates on compliance, risks and scientific evaluations of additionality and leakage in a way that fosters both cost-effectiveness and recognition of local knowledge. This can enable policy actors to establish an adaptable benchmarking system to distinguish areas of high and low risk and adjust their implementation focus with changes in risk.

Yet, many measurement and monitoring challenges remain. Protocols devised from afar tend to serve the needs of global carbon markets and lock in certain intervention pathways while excluding others [134]. These characteristics of many deforestation and restoration efforts tend to serve embedded political interests and deepen problematic power relations. In terms of policy evaluations, the measurement of spillovers and, in particular, leakage remains challenging [135]. Finally, land-based climate mitigation policies have hitherto shown little capacity for adaptation, e.g. path dependencies in the use of certain market mechanisms like payments for environmental services (PES) and many supply chain sustainability initiatives.

Designing a more just and sustainable policy mix in the land-based climate mitigation area is inherently challenged by the need to reconsider original problem definitions. Nevertheless, certain approaches can address some of the shortcomings of mainstream deforestation-reducing and restoration policy approaches. Combining features of traditional market-based approaches with bottom-up community and indigenous rights efforts can strengthen the effectiveness, legitimacy and equity of conserving and restoring forests [136–139]. Enacting a more complex policy mix may be costly and take more time to implement. Yet the enhanced legitimacy and provision of wins to multiple actor groups could help ensure the successful continuation of the policy mix over a longer period.

One notable example of a policy mix that overcomes the limitations of different individual policy approaches is the Brazilian Plan for the Prevention and Control of Deforestation in the Legal Amazon (PPCDAm), which includes a diverse set of approaches and an integrative scope across different scales and mechanisms (S6), including land planning through an expansion of protected areas, recognition of Indigenous Peoples’ territories, enhanced monitoring and enforcement and incentives and capacity building for sustainable land use [140]. This policy mix has focused both on effective and efficient reductions in deforestation using various incentives, but also prioritizes landscape diversity and elevates recognitional justice aspects through expanded areas for Indigenous stewardship of protected areas (S1, S3). In the most recent phase of PPCDAm, there are nods to more transformative approaches to supporting Indigenous rights and local communities through the co-development of sociobioeconomies (S2, S4) [46]. Monitoring is built into the policy, and different phases of PPCDAm are adapted to updated information on the success and drawbacks of earlier stages (S5).

In terms of political feasibility (S7), the success of PPCDAm may again serve as a useful lesson. Efforts to stem land cover change from agricultural expansion, for instance, very often fail because there is too much counterpressure from continued subsidies favouring expansion, often dictated by large agribusiness actors, which have similar interests and are organized into influential lobby groups [141]. In PPCDAm, however, the government continued to provide very large subsidies to agriculture for both conventional and low-carbon practices and developed flexible mechanisms for addressing gaps in legal compliance (S7). Coalition building to support indigenous and traditional communities was also a critical component of the efforts to enhance the political feasibility of PPCDAm. This example shows how policymakers were able to expand the portfolio of benefits to different actors through incremental changes, while also following a more transformative agenda.

### 4.2. Case 2: Biodiversity-friendly agriculture

Efforts to promote biodiversity-friendly agricultural systems focus on both the land-use impacts of farming systems and the biodiversity impacts of differences in management (e.g. input use, tillage, rotations and intercropping) [18,142–144]. At the agenda-setting stage, this often leads to two conceptually different strategies to reduce impacts on biodiversity: (i) increasing yields of agricultural land via intensification in efforts to ‘spare’ land for conservation and restoration leading to improved habitats in non-agricultural areas [144], and (ii) promoting agroecological practices on working farm landscapes to simultaneously support biodiversity and people, reduce carbon emissions and nutrient load within and around farms and enhance system resilience and other ecosystem services [145].

There has been considerable academic and policy debate about the best ways to balance biodiversity and food production, framing these strategies as dualistic. Indeed, the critical dimensions of agroecology are a reaction to the lack of recognitional justice inherent in much of the agenda setting around sustainability intensification and land sparing. Agroecology, therefore, explicitly elevates social, economic and political dimensions of sustainability and justice in agricultural systems in its definition and goals, encompassing ideas of political resistance to conventional agriculture and wider food systems [146]. Though academically positioned as opposing strategies, in practice, there are growing cases of national food and biodiversity strategies that focus more on recognition of land user needs and experiences to identify synergies (e.g. the Kenya National Agroecology Strategy for Food System Transformation described below).

At the policy formulation stage, policies focusing on intensification to spare land typically have a focus on the goals of efficiency (with a heavy emphasis on spatial optimization) and effectiveness (in terms of meeting biodiversity goals with the least impact to food production or vice versa). Agroecology-oriented policies, in contrast, tend to centre more on biological and biocultural diversity and justice goals, with effectiveness still in mind due to the focus on discrete food and ecological outcomes. In terms of addressing systemic issues, agroecology discourses often address consumption, questioning existing consumption trends and promoting holistic, locally grounded public food procurement programmes [147]. Yet, there are few explicitly named agroecology policies that focus on consumption changes. The agroecology movement often explicitly aims for transformative change from the onset by questioning existing institutional barriers and power structures [146], including food system concentration and lock-ins that favour corporate food actors [148,149], but policy examples of systemic change towards agroecological principles are hard to find. Intensification-focused approaches work within the status quo of conventional food systems without challenging systemic issues, though this is shifting, as evidenced by growing discussions of sustainable diets alongside sustainable intensification.

Intensification-focused policies in a land-sparing framework often formulate policy ideas with market mechanisms in mind to efficiently meet the objectives of yield improvements. These mechanisms include subsidies and credit programmes for agricultural inputs and ‘improved’ seed technologies. There is a reticence towards hard regulation, instead favouring incentives and capacity building for ‘good agricultural practices’ [150,151]. There is a heavy emphasis on technology and technological innovations that tend to resonate better with the demands of globally powerful actors to increase food production under food security narratives. Such interventions do not challenge the status quo and are often more politically palatable. Yet, they can end up locking in certain agricultural approaches that reduce adaptability and reinforce the consolidation and concentration of farmland and power in the food industry [152], favouring political interests that will continue to prioritize intensification. For example, policies to roll out irrigation programmes in the Mediterranean failed to account for land concentration and the homogenization of cropping systems, which gave rise to new system vulnerabilities [153]. Similar cases of land consolidation and smallholder exclusion have been frequently recorded to promote new seed technologies [151]. This phenomenon is a good example of why efficiency-focused efforts that may seem Pareto optimal in abstract policy discussions often violate ‘do no harm’ principles. Agroecology-oriented policies, in contrast, are inherently rooted in bottom-up community processes, including building social movements to counter dominant practices and support marginalized communities [154]. Agroecology practices themselves often strengthen community bonds, as farmers often work together sharing knowledge, seeds and labour [155].

Both intensification and agroecology struggle with taking an integrative scope. Intensification policies have undoubtedly resulted in large increases in global yields and are certainly part of the equation of meeting food demand while protecting biodiversity. Yet the cheap provision of grains and oilseeds has also helped fuel greater consumption of these products, underlying the most rapid conversion of native ecosystems to date. Conversely, there are concerns that a narrow focus on social justice in agroecological approaches can compromise biodiversity goals if they result in lower yields and agricultural expansion to meet the same level of demand. Moving forward towards a more integrative scope may entail abandoning these dichotomous trade-off framings.

At the implementation and evaluation stage, monitoring of policy outcomes is a challenge for both intensification-oriented and agroecological approaches. For instance, an agroecological farm may combine crop rotation, agroforestry and natural pest control with outcomes for nutritional diversity in diets and a safety net in the face of various types of adversity, not just the production of a singular product. The diversity and complex nature of such systems make it difficult to identify (and measure) relevant outcomes in consistent ways, and accurately and systematically monitoring agrobiodiversity targets at scale can be costly and difficult [156]. Nevertheless, such indicators can be developed, as has been done by the Tool for Agroecology Performance Evaluation developed by the FAO to monitor the impacts of agroecological transitions [157]. The Global Alliance for the Future of Food has developed a Systemic Investing Assessment tool that enables financial actors to incorporate the true environmental and social costs of food production when making investments.

Agroecology’s tension with prevailing interests means that policies that include both intensification and agroecological elements may skew actual support and investments towards intensification during implementation. In many regions, agroecological movements struggle to receive anywhere near the financial support that intensification approaches do. The path dependencies and structural lock-ins at play after centuries of extractivist land use via industrialized agri-food systems and globalized supply chains make it difficult for land-use decisions by Indigenous communities, peasants and family farmers to push the system into new trajectories [9]. There is a strong need to improve monitoring and evaluation systems to ensure that the intentions of agroecological approaches are not co-opted and to identify ways to improve implementation if this occurs. Despite these challenges, the strength of an emancipatory, decolonial agroecology approach is its ambition to weaken these relations of production over time and to create new and different coalitions and more direct relationships between actors [158]. Similarly, intensification approaches should build in more evaluation of spillovers on people and the environment to avoid unintended harmful outcomes. For example, the green revolution policies pursued in India in the early 1960s have led to dire impacts on farmers and the environment, by shifting production from a wide range of indigenous landraces to a few highly intensive commodities [159]. Yet their effects have still yet to be undone.

Promisingly, both intensification and agroecological approaches increasingly have been adapted by gradually including a more integrative focus, including calls to reduce demand for meat products and food waste, alongside production changes [144]. Here, too, there is a role for technology and for benefits to a wide range of actors through the expansion of meat substitutes. This middle way could provide a politically feasible approach to garner more support for changes in meat consumption that create benefits to some corporate food interests, while still tackling the systemic underlying problem of increasing meat demand. Intensification and agroecological approaches could be better integrated to balance justice and spillover concerns. Both approaches must be paired with strict conservation or restoration to avoid rebound effects (whereby increased profits from productivity-enhancing intensification efforts lead to more clearing) [144,160]. Yet, food and conservation policies are often separated into different policy domains, resulting in policy incoherence [161].

One example where agroecological policies have been gaining steam alongside intensification approaches is in Kenya, where several of the keystone policies and strategies, including the National Agroecology Strategy for Food System Transformation 2024−2033, have integrated agroecological principles that align with the policy roadmap outlined here. For example, agroecological efforts in Kenya recognize Indigenous traditional wisdom as a source of enhanced knowledge (P1) (e.g. in the Seed and Plant Varieties Act of 2012), rather than framing agricultural practices as outcomes of knowledge deficits. The policies also tend to embrace multiple goals (P3), including diversification, resilience and equity alongside resource efficiency, address systemic issues (P4) and have co-development (P5) and an integrative scope (P6) built in by including multiple levels of input and governance [162]. This agroecological focus in Kenya is being prioritized alongside large investments in technological improvements to increase yields. The Murango County Agroecological Policy of 2022, the first county-level policy in Kenya, also takes an integrative approach across consumption and production, focusing on environmental and human health. The National Agroecology Strategy for Food System Transformation also looks at a wide array of social, agricultural and environmental indicators to monitor policy outcomes associated with promoting agroecology (P7). Yet, the goals of these policies continue to be undermined by historical neoliberal and colonial policies that favour conventional agricultural and export-oriented production [163], reflecting the embedded political resistance to systemic change (P2).

## 5. Conclusion

In this article, we have provided a checklist of guiding policy principles to promote sustainable and just land systems using existing theory and expert knowledge. The eight principles can be summarized as follows: embrace recognitional justice (P1), be politically strategic (P2), consider multiple policy goals (P3), address systemic issues (P4), take an integrative scope (P5), foster co-development (P6), establish clear and monitorable targets (P7) and integrate diagnostic and adaptive capacities (P8). As they guide the stages of a policy cycle, these principles are designed to be used in combination, with ongoing iteration from the end of the cycle to the beginning. The stages of a policy cycle are theoretically chronological, but their main purpose is heuristic, so the checklist process should allow for alterations in timing, sequencing and layering depending on existing policies.

We then examined the extent to which existing policies align with these policy principles in two globally relevant cases: land-based climate mitigation and biodiversity-friendly agriculture. Across the two cases, we see the foundational importance of the agenda-setting stage. When the objectives of the policies (in terms of the problems they are trying to address) are formulated in narrow, top-down ways with limited actor input versus more justice-centred and politically strategic ways, the entire policy cycle is set on a trajectory that favours limited policy goals and scope and reinforces existing power structures. Efforts to co-develop the policy scope or implementation later cannot challenge the fundamental misalignment of the problem formulation.

In the climate mitigation space, we see a heavy focus at the formulation stage on top-down, incremental deforestation and restoration policies focused on effectiveness and efficiency policy goals, relying heavily on market instruments with narrow actor and spatial targets. The challenges with a non-integrative scope, combined with a failure to address systemic issues, expose these policies to low additionality, high leakage and many justice challenges. In the biodiversity-friendly farming example, there are many top-down, incremental efficiency and productivity-focused agricultural policies that reinforce existing power structures and lack adaptability to respond to the injustices in land and market access that they exacerbate.

However, in both cases, we see alternative, justice and rights-based approaches that embrace diversity as a core value gaining steam through the recognition of Indigenous peoples’ and traditional community rights and grassroots peasant agroecology movements that go beyond farmers to consumers, retailers, policymakers and scientists, among others [158]. These efforts embrace recognitional justice as a core principle and are inherently more adaptable and transformative, often making them more effective from a sustainability perspective than intensification-focused policies. Yet they are scarcely integrated into mainstream policy and often face greater political pushback. Their spillover effects also remain unclear. In the face of global environmental, development and democratic backsliding, there is an urgent need to identify more politically attuned strategies to integrate recognitional justice with early benefits to entrenched actors to avoid pushback and ratcheting down.

Notably, several examples of policy mixes that align well with most of the principles already exist. In the land-based climate mitigation case, we examined the specific example of PPCDAm in Brazil, which shows how deforestation policies, indigenous protections and transformative agendas have evolved through decades of wide-ranging advocacy campaigns, careful political negotiation and policy sequencing. This policy development process balanced various sectoral interests and has been highly successful in reducing deforestation in the Amazon [164]. In the biodiversity-friendly agriculture case, the example of Kenya’s agricultural policy frameworks shows how conventional intensification policies were over time complemented by agroecological principles, leading to a more holistic, adaptable and pluralistic approach to improving food production. In both cases, we see the most promising (and politically feasible) policy strategies where conventional policy approaches (e.g. PES and intensification subsidies) are paired with more transformative changes (e.g. changes in diets and strengthening community rights) under more comprehensive policy mix strategies.

While the complexity of issues and contexts in land systems makes a comprehensive review of sustainability and justice outcomes challenging and timely, the cases as presented here offer some initial examples about how the principles operate in practice. However, the scope of this article is exploratory, focused on developing the principles and highlighting their potential utility. Future academic work should examine the principles’ causal power in different contexts and/or use the checklist to guide ex-ante analyses of potential land system sustainability and justice under different conditions of alignment. Future questions for research include the following: What is the evidence on the degree to which existing land system policies align with the proposed principles? Are there examples of land systems which have moved from unsustainable/unjust to sustainable/just trajectories, and how was that done (in terms of alignment with the principles or not)? How does the exclusion of certain elements of the checklist affect land system outcomes? What are the main barriers and constraints to designing policy mixes that would meet the follow the principles?

The policies that we see enacted in the world are not designed through any optimization process but are the result of agreement or disagreement between different actors in the context of their different levels of authority and power [165]. Factoring in the potential for political conflicts and tensions is necessary to avoid naive use of the checklist outlined above [26]. This is of particular importance as global demands on land—for space and material commodities (food, minerals and fuel)—continue to increase and financial capital to support status quo industries increasingly outpaces the alternatives put forward by less powerful actors [46]. If left unchecked, these realities will continue to inhibit not only justice in land systems but also the robustness and effectiveness of policies aiming to address land systems sustainability.

The principles elaborated here may appear unsurprising to those already engaged in land systems research, but they are still not widely applied in real-world land systems policy. Therefore, we hope that laying the principles out in a systematic and structured way, with examples of best and worst practice, will galvanize policymakers and actors at the science–policy interface to make more use of the understanding land systems researchers have gained of what works and what does not, and for whom. Taken together, the checklist and case studies encourage policymakers and actors working at the science–policy interface to re-evaluate various stages of the policy cycle and acknowledge that certain narrow agenda-setting processes driven by limited actor interests and socio-technical and market paradigms have locked in a very limited set of policy levers with limited success [9]. By following these principles, policymakers can overcome political deadlock over intractable trade-offs and help steer land systems into new pathways that achieve greater synergies in sustainability and social justice outcomes.

## Data Availability

Supplementary material is available online [166].
